# Uterine Necrosis Associated with *Fusobacterium necrophorum* Infection

**DOI:** 10.1155/2015/934913

**Published:** 2015-04-27

**Authors:** T. Widelock, R. Elkattah, S. Gibbs, Z. Mashak, S. Mohling, S. DePasquale

**Affiliations:** ^1^Department of Obstetrics and Gynecology, University of Tennessee College of Medicine Chattanooga, 979 East Third Street, Suite C-720, Chattanooga, TN 37403, USA; ^2^Division of Gynecologic Oncology, Department of Obstetrics and Gynecology, University of Tennessee College of Medicine Chattanooga, 979 East Third Street, Suite C-720, Chattanooga, TN 37403, USA

## Abstract

*Fusobacterium necrophorum* is infrequently implicated as a pathogenic organism. When pathogenic, the typical clinical presentation is that of pharyngitis, cervical adenopathy, and unilateral thrombophlebitis of the internal jugular vein. Infections caused by *Fusobacterium necrophorum* within the fields of obstetrics and gynecology have been infrequently reported. We describe a 19-year-old woman who underwent a cesarean delivery complicated by sepsis and purulent uterine necrosis secondary to *Fusobacterium necrophorum* infection.

## 1. Case Illustration

**Figure 1:**
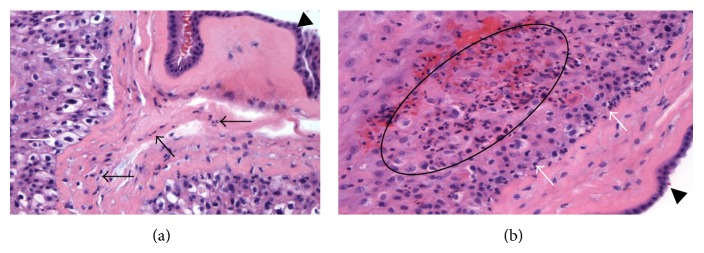
(a) Medium power magnification with Hematoxylin and Eosin staining revealing a moderately diffuse infiltrate of polymorphonuclear white blood cells (black arrows) between the amnion (arrowhead) and the chorion (white arrows). (b) Medium power magnification with Hematoxylin and Eosin staining revealing chorionitis with a dense infiltrate of polymorphonuclear white blood cells (encircled) within the chorion (white arrow). The amnion is also visible (arrowhead).

Our patient is a 19-year-old G_4_P_0212_ who presented at 33 weeks and 5 days of estimated gestational age with preterm contractions every 5–10 minutes, vaginal spotting, and pink vaginal discharge. Her pregnancy had been complicated by antiphospholipid syndrome and cervical incompetence. A McDonald cerclage was placed at 13 weeks of gestation. She denied loss of fluids upon admission and a sterile vaginal exam showed 1 cm of cervical dilation with an intact cerclage. Her past obstetrical history was noted for two prior preterm deliveries secondary to cervical incompetence. Pertinent past medical history includes asthma, depression, two prior cervical cerclages, two cesarean deliveries (one of which was a classical incision), and an appendectomy. With a concern of threatened preterm labor, the patient was admitted and started on intravenous magnesium sulfate and intramuscular antenatal corticosteroids for fetal lung maturity. She was also given vancomycin for group B-streptococcal prophylaxis as she was allergic to penicillin. Uterine contractility increased in frequency thereafter and the patient then developed leakage of fluids and was ruled in for preterm premature rupture of membranes. Expectant management with latency antibiotics was followed. By the second day after admission the patient continued to leak clear vaginal fluid but progressed to have more frequent uterine contractions. A thick, foul-smelling yellow discharge emanated from the external cervical os and she developed uterine tenderness. At this point, the patient was stable and fetal heart tones were reassuring but the decision to remove the cerclage was made in light of impending chorioamnionitis with the newly diagnosed uterine tenderness. Accordingly, magnesium sulfate was discontinued, intravenous gentamicin was added, and the cerclage was removed. A sterile speculum exam revealed a visually 3 cm dilated cervix prompting delivery via repeat cesarean section. Precesarean white blood cell count was 25400 cells/mm^3^ with a 90% left-shift. She underwent an uneventful cesarean delivery of a female infant weighing 2092 grams with Apgar scores of 7 and 8 at one and five minutes, respectively. Umbilical cord arterial and venous blood pHs were 7.19 and 7.26. Arterial blood gases were as follows: Pco_2_ = 72 mmHg, Po_2_ = 45 mmHg, HCO3− = 23 mmol/L, and base excess = 1. The neonate was started on prophylactic broad-spectrum antibiotics and had an uneventful course. The neonate's complete blood count at the time of birth revealed the following parameters: white cell count of 15700 cells/mm^3^, hemoglobin of 20.3 g/dL, hematocrit of 61.5%, and platelet count of 308000/mm^3^, and the blood cultures were negative for any bacterial growth. Placental bacterial cultures from both fetal and maternal surfaces were collected. The maternal interface of the placenta cultured methicillin resistant staphylococcus aureus (MRSA) and on pathologic evaluation revealed moderate chorionitis (Figure 1).

**Figure 2:**
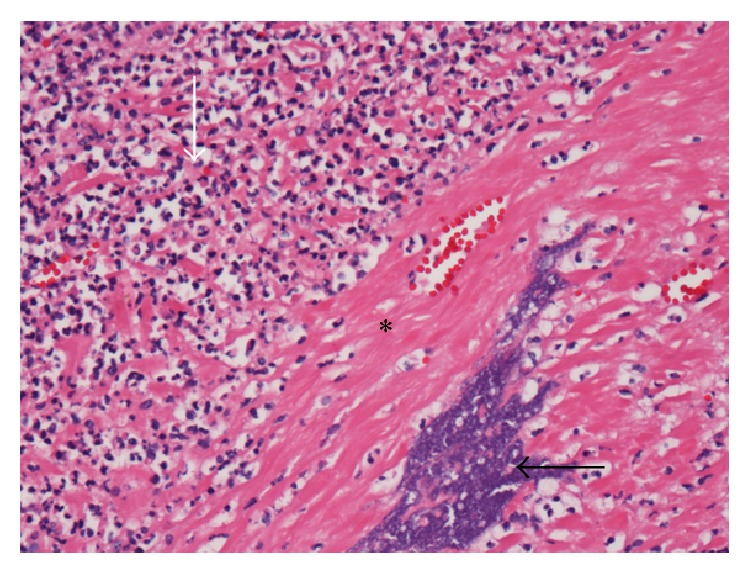
High power magnification with Hematoxylin and Eosin staining revealing a diffuse infiltrate of polymorphonuclear white blood cells (white arrow), necrotic anuclear smooth muscle cells (asterisk), and purulence (black arrow).

**Figure 3:**
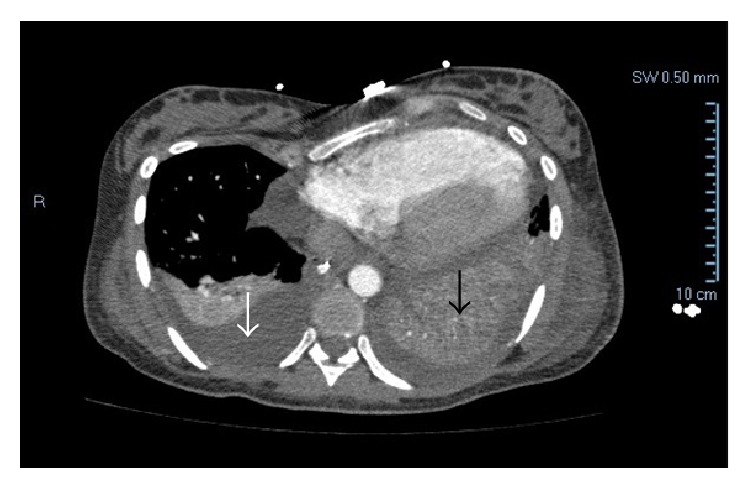
Axial chest computed tomography scan with intravenous contrast revealing right pleural effusion (white arrow) and left pleural effusion with consolidation (black arrow).

On the first postoperative day, the patient complained of uncontrollable moderate-to-severe abdominal and pelvic pain that worsened with deep inspiration. She progressed to develop marked tachycardia of 160 beats/min, tachypnea with 25 breaths/min, worsening chest pain, and shortness of breath. On physical exam her abdomen was distended and soft and foul-smelling vaginal discharge was noted. A computed tomography (CT) angiogram was performed and ruled out pulmonary embolus as a cause of her symptoms. Her symptoms worsened dramatically with further abdominal distension and an increase in her vaginal discharge. She developed a temperature of 102°F, chills, sustained tachycardia, tachypnea, and a white blood cell count of 2500 cells/mm^3^ with a 90% left-shift. Sepsis was a major concern at this point and blood cultures were obtained. Repeat CT imaging with intravenous contrast revealed an enlarged postpartum uterus, free air in the abdomen, copious fluid in the pelvis, and diffuse mesenteric edema indicative of peritoneal inflammation. She underwent an exploratory laparotomy. The patient's abdomen was noted to be full of purulent discharge and the uterus was necrotic with overlying fibrinous exudates. An estimated 700 mL of purulent discharge was evacuated and sent for cultures followed by a hysterectomy as the uterus was necrotic and was presumed to be the focus of infection. The procedure was uncomplicated. The entire small bowel was run and noted to be normal. The surgery was concluded with copious irrigation. She was then transferred to the intensive care unit and started on intravenous vancomycin and meropenem. Her pathology report noted diffuse polymorphonuclear infiltrates, necrotic myometrial tissue, and purulence on high power microscopy (Figure 2). Both blood and purulent discharge cultures grew* Fusobacterium necrophorum* and the antibiotics were continued for an additional 48 hours. By hospital day 14, the patient was in a stable recovery but had complained of nausea and occasional vomiting and difficulty in breathing. She had subsequent bilateral pleural effusions, left lung pneumothorax, left lung consolidation (Figure 3), acute urinary retention, renal failure, multiple abdominal and pelvic abscesses, and persistent nausea and vomiting. The patient had a nasogastric tube placed and underwent multiple drainage procedures for the pelvic and abdominal abscesses and had a chest tube placed for her left pulmonary pneumothorax and consolidation that subsequently tested positive for FN. At this point, vancomycin and meropenem were restarted. Repeat CT imaging of the chest, abdomen, and pelvis showed stable fluid collections thereafter and clinical improvement followed. The patient's last febrile episode was on hospital day 25. Renal function, nausea, and vomiting improved slowly and her bowel function returned to normal thereafter. The patient was eventually discharged from the hospital on day 34 from admission and was scheduled for a 14-day course of intravenous ertapenem. Her recovery was otherwise uneventful thereafter. The neonate remained in the intensive care unit for 19 days and was then discharged in a stable condition.

## 2. Background

*Fusobacterium necrophorum* (FN) is a pleomorphic Gram-negative, non-spore-forming obligate anaerobic coccobacillus [1–3]. It is associated with localized abscesses, throat infections, and systemic life-threatening disease [1]. It is a normal inhabitant of the oral cavity and the vagina [2–4]. FN infections have been well documented for over a century with the earliest descriptions of FN in the female genital tract by Halle in 1898 [4]. Only a few hundred cases have been described in the medical literature and the vast majority of these cases are nongynecologic [5]. Of the two subspecies of FN, Biovar B is the main human pathogen. Suggested virulence factors include cell wall endotoxic lipopolysaccharide, haemagglutinin, and hemolysin, but little is known about the actual role these have in the pathogenesis of FN [1].

### 2.1. Clinical Presentation

Albeit rare, the typical clinical picture of FN infection as described by Lemierre [5–7] is a young adult or adolescent with a history of pharyngitis followed by fever and subsequently rigors that occur 4-5 days after the pharyngitis symptoms. This is usually accompanied by cervical lymphadenopathy and unilateral thrombophlebitis of the internal jugular vein [7]. It is historically referred to as the “forgotten disease” [1]. Reported potential foci of FN infection include inflammatory lesions of the nasopharynx, mouth and jaw infections, otitis media, mastoiditis, purulent endometritis, appendicitis, and urinary passages [1]. These infections may be complicated by distant hematogenous spread to joints, lungs, soft tissues, and liver [5] causing septic arthritis, pulmonary effusions/abscesses, soft tissue abscesses, and liver abscesses, respectively [1, 5]. Lung involvement is almost always present. This is thought to be attributed to alterations in normal tissue architecture resulting in invasive FN and is thought to represent a superinfection, as it is rarely pathogenic and invasive but rather is thought to be a commensal and opportunistic bacterium [1, 3, 5].

Reports of gynecologic infections attributed to FN include intrauterine device-associated endometritis and ascending salpingitis with subsequent and rapid fatal peritonitis, particularly those associated with the discontinued Dalkon shield and the Lippes loop [2]. Vertical transmission to the upper genital tract along the Dalkon shield thread “tail” explains the mechanism by which this occurred [2] as 86% of cultures obtained from Dalkon shield tails tested positive for anaerobic bacteria including FN [8]. Systemic FN infections arising from the human female genital tract are exceedingly rare [5] and most reported cases occurred in the postpartum or postabortion period in addition to few reports associated with the use of intrauterine devices and tubo-ovarian abscesses [2, 5, 9, 10]. Cervical conization complicated by FN sepsis with pulmonary and hepatic abscesses has also been reported [5]. FN infection has also been associated with the postcesarean development of a vesicouterine fistula within 5 days of delivery [3]. In the obstetrical realm, FN and other anaerobes have been implicated in preterm labor with intact membranes secondary to its high phospholipase A2 activity [11]. Transient bacteremia of FN has been found to be related to preterm labor, chorioamnionitis, and intrauterine fetal demise [10].

### 2.2. Demographic Info

The overall annual incidence of FN infection has been reported to be 0.9–2.3 cases per million with a modest increase with time [1]. Invasive FN favors males and young adults within the ages of 16–23 [1]. There is a 1.5–3 : 1 ratio of male to female infections due to invasive FN, and this difference is not understood [1]. Significant mortality from untreated invasive FN can reach 90%; however with the discovery of antibiotics, present day mortality estimates of up to 26% have been reported [1]. Mortality from FN infections not originating from the head and neck (non-Lemierre related) is 26% [12].

### 2.3. Diagnosis

In order to diagnose an invasive FN infection, an anaerobic blood culture should be obtained. Conventional methods would require subculturing to nutrient enriched blood agar such as Fastidious Anaerobe Agar (FAA Lab M Ltd., Bury, UK) [1]. Gram-staining aids in the diagnosis as FN typically appears as a short coccobacillus with occasional long filamentous forms [1]. Gas liquid chromatography that analyzes volatile fatty acid profiles is also helpful [1].

### 2.4. Treatment

Most treatment regimens are prolonged ones [12] and fevers may take several weeks to resolve with therapy [1]. To date, there is no reported resistance to metronidazole, amoxicillin-clavulanate, chloramphenicol, cefoxitin, clindamycin, or imipenem; however it is not susceptible to macrolides and quinolones [12] with a reported 15% resistance to erythromycin [1]. FN responds well to antibiotics with anaerobic coverage and beta lactamase resistance and is rarely resistant to penicillin [12]. Previously used antibiotics with success include ticarcillin-clavulanate, ampicillin-sulbactam, amoxicillin-clavulanate, cefoxitin, and metronidazole [6]. Intravenous therapy is recommended up until when patients defervesce or neck swelling resolves—whichever comes first—followed by additional oral therapy for up to 4 weeks after this initial phase [6]. Most authors recommend combination of penicillin and metronidazole for several weeks. IV Pen G 2–5 million units QID + flagyl 500 TID × 2 weeks followed by PO amoxicillin 500 mg TID + flagyl 500 mg TID × several weeks until inflammation resolves [12]. In individuals allergic to penicillin, clindamycin 1800 mg divided into 3-4 doses daily is an appropriate alternative.

Surgical drainage of abscesses and fluid collections is associated with improved outcomes and is thus highly recommended [1, 12]. FN can cause a serious and worsening infection in young healthy adults that progresses quickly and has the potential to cause significant morbidity as had happened in our case with associated pulmonary effusions, septic shock, and acute renal failure [1, 5]. FN can also lead to pneumonia and death [1, 5]. Repeated CT scans of the chest are required for early detection of chest related complications and for drainage of empyemas and effusions [12].

## 3. Discussion

In the realm of obstetrics and gynecology,* F. necrophorum* has rarely been implicated as a major source of infection. There are several sporadic reports associating FN with female genital tract infections, morbidity, and mortality. Systemic FN infections arising from the human female genital tract are exceedingly rare [5] and most reported cases occur in the postpartum or postabortion period in addition to few reports associated with the use of intrauterine devices and tubo-ovarian abscesses [2, 5, 9, 10]. Our literature review included an extensive search within PubMed/Medline database and open-sources using the following search terms: “*Fusobacterium necrophorum*,” “*Fusobacteria*,” “*Fusobacterium*,” “*bacterial infection*,” and “*obstetrics*,” “*gynecology*,” “*cerclage*,” and “*uterine necrosis*.” We believe that this is the first case of uterine necrosis secondary to an FN infection.

In this report, the patient resembled the typical young patient that Lemierre described [6, 12]; however the typical presentation was not that of upper respiratory tract involvement, cervical adenopathy, and unilateral thrombophlebitis of the internal jugular vein. With the genital tract of females as a potential focus of infection, this patient became septic and developed systemic infection secondary to a fulminant FN infection that leads to purulent endometritis and eventually uterine necrosis. The intra-abdominal purulence that was removed at the time of laparotomy tested positive for FN and included approximately 700 mL of fluid. Her infection was complicated by lung involvement as indicated by the worsening left pleural effusion and this is in line with the known natural history of systemic FN infections and pulmonary involvement. The pulmonary infiltrates drained from the patient's chest tube also tested positive for FN, thus confirming distant hematogenous spread.

There are several factors that could have contributed to the virulence of this otherwise nonpathogenic bacterium. In previous reports, this virulence has been attributed to alterations in normal tissue architecture secondary to primary infections with pathogens other than FN and thus FN represents a superinfection. This may have been the case in this report as the maternal interface placental culture tested positive for MRSA prior to the actual fulminant FN infection. Systemic steroid exposure may have also contributed to this virulence. Similar to the vesicouterine fistula reported by Baker [3], our patient had received antenatal corticosteroids to accelerate fetal lung maturity three days prior to developing any systemic symptoms and this may have contributed to the virulence of FN. Ruptured membranes also facilitate the vertical transmission of vaginal pathogens. Lastly, we suggest a role of the cerclage in the vertical transmission of FN within the cervical stroma and likely towards the intrauterine cavity in a “wick” mechanism similar to that of previously reported intrauterine devices [2, 8] as the patient had clearly been in active labor prior to removal of the cerclage and this may have compromised the cervical integrity and led to enhanced accessibility of FN to the blood stream similar to what was reported by Treszezamsky et al. [5]. This finding cannot be confirmed as the cerclage material was discarded and not cultured at the time of removal.

As outcomes improve with surgical drainage of fluid collections and pus [1, 12], our patient showed prompt improvement in her pulmonary status and her abdominal pain. This followed interventional radiologic drainage of perisplenic and pelvic fluid collections after hysterectomy. She also had a left chest tube placed for pleural effusions and consolidations. This leads to dramatic improvements in her pulmonary status with breathing rates normalizing, minimal abdominal and pelvic pain, and decreased nausea and vomiting followed by a return of normal bowel function and appetite.

Similar to other reports of FN infections, febrile illness was present intermittently through days 3 to 25 of our patient's hospital stay and the patient required an extended period of intravenous antibiotics after discharge from the hospital. She remained afebrile thereafter and had an uneventful and complete clinical recovery.

## 4. Conclusion

*Fusobacterium necrophorum* is a potentially serious infection in postpartum patients. The triad of purulence, abdominal pain and fever in addition to lung involvement should alert the obstetrician gynecologist to this potential pathogen despite the lack of any pathognomonic signs of this rare infection. The role of cerclage and FN infection is unclear and warrants further investigation. With prompt diagnosis and extended antibiotic therapy, morbidity and mortality following a FN infection may be averted.
